# Potentially Inappropriate Prescribing Identified Using STOPP/START Version 3 in Geriatric Patients and Comparison with Version 2: A Cross-Sectional Study

**DOI:** 10.3390/jcm13206043

**Published:** 2024-10-10

**Authors:** Mikołaj Szoszkiewicz, Ewa Deskur-Śmielecka, Arkadiusz Styszyński, Zofia Urbańska, Agnieszka Neumann-Podczaska, Katarzyna Wieczorowska-Tobis

**Affiliations:** 1 Geriatric Unit, Department of Palliative Medicine, Poznan University of Medical Sciences, 61-245 Poznan, Poland; 2 Student Scientific Section of Department of Palliative Medicine, Poznan University of Medical Sciences, 61-245 Poznan, Poland

**Keywords:** potentially inappropriate medications, potential prescribing omissions, explicit tools, STOPP/START criteria

## Abstract

**Background:** Multimorbidity, polypharmacy, and inappropriate prescribing are significant challenges in the geriatric population. Tools such as the Beers List, FORTA, and STOPP/START criteria have been developed to identify potentially inappropriate prescribing (PIP). STOPP/START criteria detect both potentially inappropriate medications (PIMs) and potential prescribing omissions (PPOs). The latest, third version of STOPP/START criteria expands the tool, based on the growing literature. The study aimed to evaluate the prevalence of PIP and the number of PIP per person identified by STOPP/START version 3 and to compare it to the previous version. **Methods:** This retrospective, cross-sectional study enrolled one hundred geriatric patients with polypharmacy from two day-care centers for partially dependent people in Poland. Collected data included demographic and medical data. STOPP/START version 3 was used to identify potentially inappropriate prescribing, whereas the previous version served as a reference. **Results:** STOPP version 3 detected at least one PIM in 73% of the study group, a significantly higher result than that for version 2 (56%). STOPP version 3 identified more PIMs per person than the previous version. Similarly, START version 3 had a significantly higher prevalence of PPOs (74% vs. 57%) and a higher number of PPOs per person than the previous version. The newly formed STOPP criteria with high prevalence were those regarding NSAIDs, including aspirin in cardiovascular indications. Frequent PPOs regarding newly formed START criteria were the lack of osmotic laxatives for chronic constipation, the lack of mineralocorticoid receptor antagonists, and SGLT-2 inhibitors in heart failure. **Conclusions:** This study showed the high effectiveness of the STOPP/START version 3 criteria in identifying potentially inappropriate prescribing, with a higher detection rate than version 2.

## 1. Introduction

The World Health Organization defines multimorbidity as the presence of two or more chronic conditions in one patient [1]. Multimorbidity, a prevalent phenomenon among older people, is associated with a higher risk of polypharmacy and inappropriate medication. Polypharmacy is a growing concern due to the increased risk of prescribing problems, adverse drug reactions, drug–drug interactions, and drug–disease interactions [2,3].

Criteria for potentially inappropriate prescribing (PIP) have been developed to reduce the number of drug-related problems. Multiple tools supporting the optimization of pharmacotherapy in geriatric patients have been created; for example, there are questionnaires, such as MAI (the Medication Appropriateness Index), lists of potentially harmful medications, e.g., the Beers list, and other instruments available that also cover issues of undertreatment, such as FORTA (Fit fOR The Aged) and STOPP (Screening Tool of Older Person’s Prescriptions)/START (Screening Tool to Alert doctors to Right Treatment) [4,5,6,7]. These tools have been categorized as PILA (patient-in-focus listing approach), DOLA (drug-oriented listing approach), and DOLA+ (drug-oriented listing approach but indication required) [6]. Analyses of 58 explicit tools revealed limited overlap between them, which underlines the need to standardize such criteria [8]. Experts from eight European countries validated the STOPP/START criteria, making it suitable for analyzing pharmacotherapy in our study.

**Figure 1:**
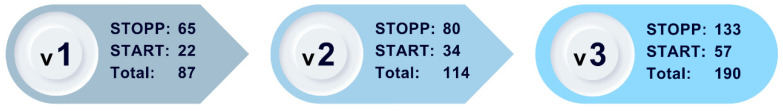
Comparison of the net number of criteria between three versions of the STOPP/START.

STOPP/START identifies both potentially inappropriate medications (PIMs) and potential prescribing omissions (PPOs), making it a useful tool for optimizing pharmacological treatment in older individuals, classified as PILA. However, when considered separately, the STOPP section is considered to be DOLA+ [7,9]. These criteria are organized into sections representing different physiological systems, making them easily applicable and widely used in clinical practice [10]. The original STOPP/START criteria were published in 2008, followed by the second version in 2014, containing more criteria (Figure 1) [11]. The effectiveness of these criteria has been compared a few times, and the second version has usually shown a higher detection rate of PIP. It appears that the prevalence of PIMs is significantly higher for version 2. However, there are conflicting results concerning the superiority of the second version in finding PPOs [12,13,14].

The third version of STOPP/START criteria, published in 2023, contains 133 STOPPs and 57 STARTs. The difference in the net number of criteria between STOPP/START version 3 and version 2 is approximately twice as large as that between the first two versions, with increases of 67% and 31%, respectively (Figure 1) [7,11,15]. The authors stated that this expansion was the result of both a growth in published evidence related to pharmacotherapy in older people and the availability of new medications [15]. In addition to adding new criteria, the authors of the third version dropped six criteria and modified a considerable amount of the rest, compared to the second version [16]. Some clinicians claim that such an extension will influence usability in daily practice [16,17]. Thus, we wanted to assess the influence of the described modifications on the effectiveness of PIP detection. Our study aims to evaluate the prevalence of PIP and the number of PIP per person identified by STOPP/START version 3 and to compare the new criteria with the previous version.

## 2. Materials and Methods

### 2.1. Participants and Procedure

The retrospective, cross-sectional study was conducted in two day-care centers for partially dependent elderly subjects in Poland in 2022 and 2023. The inclusion criteria for the day-care centers were as follows: age ≥ 60, Barthel Index between 40 and 65 points, and hospitalization in the preceding 12 months. The Barthel Index is a scale used to measure performance in activities of daily living, with the range from 40 to 65 points indicating partially dependent patients [18]. Seniors taking at least 5 medications daily on admission to a day-care center were selected for our analysis. For pragmatic reasons, the study included the last 100 consecutively admitted patients with polypharmacy to day-care centers without prior sample size calculation. Geriatricians examined patients and collected the following information: age, sex, medical history, diagnoses, current medication list, biochemical results (electrolytes, creatinine level, and complete blood count) and vaccination history (pneumococcal, COVID-19, and influenza vaccinations). Geriatricians actively asked about major geriatric syndromes, including falls, urinary incontinence, constipation, dementia, depression, and sleep disorders. Data on admission were analyzed in this study. The Charlson Comorbidity Index (CCI) was calculated to assess comorbidity burden. The presence of cognitive impairment was assessed based on Mini-Mental State Examination (a score less than 24 points indicated impairment) and/or the presence of dementia in previous history. Data were collected from electronic medical records for research purposes between October and November 2023, excluding any personal identifiers.

### 2.2. Evaluation of PIMs and PPOs

PIP was assessed using the third version (v3) of STOPP/START. The physician assessed PIMs and PPOs and discussed any concerns with two other geriatricians. The prevalence of PIMs was defined as the number of patients with at least one PIM detected by STOPP. The prevalence of PPOs was defined as the number of patients with at least one PPO detected by START. Of the 133 STOPP v3 criteria, 123 (92.5%) were used to assess PIMs and 46 out of 57 START v3 criteria (80.7%) were used to evaluate PPOs. The effectiveness of the new criteria was compared to the STOPP/START version 2 (v2). Of the 80 STOPP v2 criteria, 73 (91.3%) were used to assess PIMs and 29 out of 34 START v2 criteria (85.3%) were included to evaluate PPOs.

The total list of STOPP/START criteria we did not use due to a lack of specific data is placed in the Supplementary Materials. Comparative revision was conducted to exclude potential bias due to differences in the number of used criteria in both versions. Among the unused STOPP criteria, seven were present in both versions, three were newly formed in STOPP v3, and none were present only in STOPP v2. Among the unused START criteria, four were present in both versions and seven had been newly created for START v3. Only one not-used START criterion was exclusively present in START v2 (C4—topical prostaglandin, prostamide, or beta-blocker for primary open-angle glaucoma). This criterion was excluded because of insufficient data about the history of ophthalmic diseases in an electronic database.

The criteria STOPP A1 (any drug prescribed without an evidence-based clinical indication), STOPP A2 (any drug prescribed beyond the recommended duration, where the treatment duration is well defined), and START A3 (any drug clearly indicated and considered appropriate in the particular clinical context) were not analyzed due to the ambiguity of criteria allowing for different interpretations and the lack of necessary medical outcomes, for example, the level of serum magnesium for widespread magnesium preparations. The criterion STOPP H3 in version 3 (vitamin D supplement in older people with a confirmed 25-hydroxycholecalciferol deficiency) and the equivalent in the second version were not analyzed due to their incompatibility with national guidelines, which recommend senior cholecalciferol supplementation without a screening of serum 25(OH)D for the entire population [19]. The vaccine criteria were analyzed separately because the vaccination history was available for only 82% of the study population. Additionally, cumulative counting with other START criteria may misrepresent the results due to the low level of pneumococcal and influenza vaccination in the Polish population. Geriatricians actively asked about pneumococcal, influenza, and SARS-CoV2 vaccines. They did not ask about the varicella vaccine; thus, the START L3 (varicella-zoster vaccine according to national guidelines) criterion was excluded.

### 2.3. Statistical Analysis

Descriptive statistics were used to describe the study population and the number of PIMs and PPOs detected by two STOPP/START criteria versions. The normality of the distribution was checked using the Shapiro–Wilk test. The data are presented as a mean ± standard deviation for normally distributed quantitative parameters and a median with interquartile range (IQR) for non-normally distributed parameters. McNemar’s test was used to compare the prevalence of PIP detected by two versions of the criteria. Due to the non-normally distributed variables, the Mann–Whitney U test was used to compare the number of PIP detected by the two criteria versions. The concordance between the two STOPP/START versions was analyzed using kappa tests for nominal variables (where values of kappa of less than 0.4 indicated poor agreement, values between 0.4 and 0.6 indicated moderate agreement, values between 0.6 and 0.8 indicated substantial agreement, and kappa values above 0.8 indicated excellent agreement). The correlation between the two STOPP/START versions for continuous variables was analyzed using Kendall’s test (where values of Kendall’s test of less than 0.3 indicated weak positive correlation, values between 0.3 and 0.5 indicated moderate positive correlation, and values above 0.5 indicated strong positive correlation). Gender, multimorbidity burden based on the CCI, and the presence of cognitive impairment were included in the multiple logistic regression analysis to determine factors associated with the presence of PIMs, as detected by STOPP, and of PPOs, according to the START criteria. Statistical analysis was performed using PQStat Software (2023, PQStat v.1.8.6.116).

## 3. Results

### 3.1. Description of the Study Sample

A total of 100 patients taking at least five medicines were included in the study. The participants’ mean age (±SD) was 79.3 (±7.7), varying from 63 to 97 years. Women accounted for 64% of patients. The median of CCI was four (IQR 4–5.25). The total number of medicines ranged from five to nineteen, with a median of nine (IQR: 7–11). Of the patients, 55 received between five and nine medicines and 45 subjects had at least ten drugs. In total, 957 medication instances were analyzed.

### 3.2. STOPP

STOPP v3 identified 179 PIMs in 73% of the study sample (Table 1). Of the patients, 25% were prescribed one potentially inappropriate medicine, 24% were prescribed two, 10% were prescribed three, and 14% were prescribed four or more such medications. The majority of PIMs was associated with the coagulation system, which accounted for 21% of PIMs, followed closely by the central nervous system (CNS) with 20%, and the cardiovascular system with 14%. The most frequent PIMs were the use of regular opioids without concomitant laxatives (7.8%), drugs causing constipation in patients with constipation (7.3%), long-term opioids for osteoarthritis (6.2%), aspirin for primary prevention of cardiovascular disease (6.2%), long-term aspirin at doses greater than 100 mg per day (5.6%), and the use of benzodiazepines for more than four weeks (5.6%). Considering drug classes, nonsteroidal anti-inflammatory drugs (NSAIDs) accounted for 24% of PIMs, of which 15.6% applied to aspirin in cardiovascular disorders. Opioids constituted 18.4% of PIMs, whereas benzodiazepines comprised 8.9% of PIMs.

STOPP v3 detected 73.8% more PIMs than version 2. The updated version found more PIMs in nine out of thirteen physiological system categories. The most notable differences were observed for the coagulation system (24 more instances), the cardiovascular system (13 more instances), the musculoskeletal system (11 more instances), and the CNS (11 more instances). Of the eleven criteria with a prevalence of at least 5%, six were absent in STOPP v2.

The number of PIMs per person identified by STOPP v3, with a median of two, was significantly higher than those detected by START v2, with a median of one. The Kendall’s test value was 0.50, suggesting a moderate to strong positive correlation between the two versions of criteria. The prevalence of PIMs identified by STOPP v3 and the previous version was 73% and 56%, respectively. Statistical analyses showed a significant difference, whereas the kappa statistic was 0.6, indicating moderate to strong concordance (Table 2).

### 3.3. START

START v3 identified 167 PPOs in 74% of the study sample (Table 3). Of the subjects, 22% had one PPO, 24% had two, 16% had three, and 12% had four or more omissions. The majority of PPOs were associated with the cardiovascular and gastrointestinal systems, accounting for 37.7% and 25.2%, respectively. The most common PPO criteria were osmotic laxatives for chronic constipation (18%), SGLT-2 inhibitors in heart failure (13.2%), mineralocorticoid receptor antagonists in heart failure (7.8%), and statin therapy with a history of cardiovascular disease (6%). Considering drug classes, laxatives accounted for 26.4%, the highest proportion of PPOs. The next most common drug classes included the following cardiovascular medicines: SGLT-2 inhibitors (13.2%), mineralocorticoid receptor antagonists (7.8%), ACE inhibitors (6.6%), and statins (6%). As described above, we analyzed the vaccines criteria for 82% of the study sample. Only one subject reported receiving the pneumococcal vaccine and five reported receiving the influenza vaccine. In contrast, 71 patients were vaccinated against SARS-CoV2.

START v3 detected 75.8% more PPOs than version 2. Two of the eight categories showed an increase: the gastrointestinal system (39 more instances) and the cardiovascular system (25 more instances). Two categories, the coagulation and the renal systems, were added to the updated START version. From among the twelve START v3 criteria with a prevalence of at least 5%, five were absent in version 2.

The number of PPOs per person identified by START v3, with a median of two, was significantly higher than those detected by START v2, with a median of one. The result of Kendall’s test was 0.48, suggesting a moderate to strong positive correlation between criteria. The prevalence of PPOs detected by START v3 was 74%, whereas the prevalence detected by START v2 was 57%. McNemar’s test confirmed a significant difference; the kappa statistic was 0.64 and showed a substantial agreement between the two criteria versions (Table 2).

### 3.4. Analysis of Factors Associated with Potentially Inappropriate Prescribing

The influence of cognitive impairment, comorbidity burden (based on CCI), and gender were analyzed in the multivariable logistic regression. The association between those factors and the prevalence of PIMs or PPOs was checked. Regarding STOPP v3 and STOPP v2, none of these factors affected the presence of PIMs. Comorbidity burden was an independent factor predisposing to PPOs detected by START v3. Although, within START v2, the odds ratio of the comorbidity burden amounted to 1.13, this effect was not significant (95% CI 0.87–1.46). Table 4 presents the detailed results of multivariable regression.

## 4. Discussion

To the best of our knowledge, this is the first study evaluating the prevalence of PIP and the number of PIP per person identified by STOPP/START version 3 criteria. STOPP/START v3 consists of 190 criteria which cover various clinical issues. The net number of criteria has been extended relevantly, with a net increase of 67% compared with the previous version. According to the authors, this reflects the growth in evidence in the literature regarding drug-related problems [15]. STOPP v3 and START v3 detected at least one PIM/PPO in a substantial proportion of our study population (73% and 74%) with a median of two PIMs/PPOs per patient. The previous STOPP/START version 2 is well-described in the literature and served as a reference. We found that the new version identified at least one incidence of PIP in a higher proportion of the population and more PIP per person than the previous one. The above conclusion applies to both PIMs detected by STOPP and PPOs identified by START criteria.

STOPP v2 identified at least one PIM in 56% of patients, with a median of one PIM per patient. Our findings about STOPP v2 correspond with the existing literature; according to a systematic review, the average prevalence of PIMs detected by STOPP v2 was 42.8% for community patients [20]. START v2 detected at least one PPO in 57% of patients, with a median of one PPO per person. Considering that we analyzed the vaccination section separately, these results correspond well with the literature [9,12,13]. However, differences in our sample selection (polypharmacy as an inclusion factor) and the enabling of partially dependent persons limit such comparisons. Comorbidity burden has previously been identified as a predictor of PPOs detected by START v3. Previous studies described similar associations regarding START v2 [12,13]. In our study, the positive odds ratio of the CCI in START v2 was not significant, possibly due to the limited sample size.

For both STOPP and START, the concordance between PIP detected by the new and the previous version was moderate to strong, according to kappa statistics. Similarly, the correlation between the number of PIMs and the number of PPOs identified by both versions was moderate to strong, based on the results of the Kendall’s test. The authors of the tool removed six STOPP/START v2 criteria from the updated version [15,21]. We analyzed five of them; none exceeded a prevalence of 5% in our study sample. Eleven of the twenty-three criteria with a prevalence of at least 5% were newly formed.

The STOPP/START v3 criteria contain relevant modifications regarding NSAIDs. Two criteria describe aspirin in cardiovascular indications (C1: “Long-term aspirin at doses greater than 100 mg per day.”; C16: “Aspirin for primary prevention of cardiovascular disease”). In our study, aspirin was used for primary prevention of cardiovascular disease by eleven patients, whereas ten received 150 mg daily in the long term. The argument for the implementation of both criteria is an increased risk of bleeding events. Complex metanalyses revealed that, although aspirin in primary prevention was associated with reducing myocardial infarctions and strokes, major bleeding events (including gastrointestinal bleeding) balanced potential benefits [22,23]. Another new criterion involves the use of proton pump inhibitor during the use of NSAIDs to prevent gastrointestinal adverse effects (F3: “Proton pump inhibitor with short-term (<2 weeks) or longer-term (>2 weeks) NSAID.”) [24]. A positive association between nonadherence to the STOPP/START v2 criteria and gastrointestinal bleeding was recently proven [25]. We assume that the third version may increase the relevance of this association. Another criterion limiting to COX-2-selective NSAIDs within STOPP v2 was extended to all NSAIDs, regardless of COX selectivity, in version three (B17: “Long-term systemic, i.e., non-topical NSAIDs with a known history of coronary, cerebral or peripheral vascular disease” in STOPP v3). Due to the aforementioned changes, we noticed four NSAID-related criteria with a prevalence of at least 5% in version 3, compared to none in version 2. Aspirin and other NSAIDs accounted for 24% of identified PIMs by STOPP/START v3.

STOPP/START version 3 is more focused on major geriatric syndromes than the previous version. The authors address more criteria describing patients with dementia, urinary incontinence, and chronic constipation. The section involving drugs that predictably increase the risk of falls is extended. Antiepileptic, first-generation antihistamine, opioid, antidepressant, and antimuscarinic drugs are added to previously present benzodiazepines, neuroleptics, vasodilators, and Z-drugs. In our study, the newly formed criterion concerning antidepressants (K8: Antidepressants in patients with recurrent falls) exceeds a prevalence of 5% within the studied population. Many studies proved a distinct association between an increased fall risk and antidepressants usage [26,27]. Criteria within falls-section are now clarified; they apply to “patients with recurrent falls”. Recurrent falls classify patients as a high risk for subsequent events. However, “World guidelines for falls prevention and management for older adults” recommend, that fall severity, context, characteristics, and consequences are as relevant as fall frequency [28]. It may be reasonable to extend the criteria within the fall section to those aspects.

Several other newly formed STOPP/START criteria markedly impacted cumulative results. Long-term opioids for osteoarthritis were a common PIM in our study (11% of patients). Recent studies revealed that only a minority of carefully selected patients with osteoarthritis profit from long-term opioid use [29]. Omissions related to cardiovascular drugs (statin—10% of patients, ACE inhibitors—9% of patients, beta-blockers—6% of patients) were frequent within STOPP v2. When the new version was employed, the lack of these drugs remained common PPOs. In addition, there were two other more prevalent cardiovascular PPOs—the lack of mineralocorticoid receptor antagonists (13% of patients) and SGLT-2 inhibitors (22% of patients) in heart failure. Particularly frequent omissions related to SGLT-2 inhibitors may result from the recent extension of indications for its use in heart failure [30]. In Poland, an additional obstacle to the spread of SGLT-2 inhibitors is their high price. The most common PPO was the lack of osmotic laxatives for chronic constipation, prevalent in 30% of the studied population. Constipation affects 30–40% of the geriatric population worldwide [31]. Our study indicates undertreatment of this condition in the studied population, even in patients receiving opioids. Interestingly, three criteria (the lack of mineralocorticoid receptor antagonists and SGLT-2 inhibitors in heart failure, as well as the lack of osmotic laxatives for chronic constipation) accounted for 90.3% of additional omissions identified by the new version of the START criteria.

This study is an important voice in the ongoing STOPP/START criteria discussion. The third version of this tool represents a substantial increase, with the net number of criteria increasing by over two compared to the original version from 2008. Multiple clinicians and clinical pharmacists argued that 190 indications make STOPP/START criteria demanding and time-consuming in daily practice [16,17]. Our study shows that relying on the older versions of the START/STOPP criteria would lead to missing important aspects of correct pharmacotherapy. The authors of the tool reflect on challenging concerns by highlighting a strong need to create clinical decision support software (CDSS) [32]. Two multicenter trials have examined the effect of CDSS when implicating STOPP/START version 2 so far. The implementation of CDSS advice was suboptimal (15% and 62%) in both studies [33,34]. Moreover, a recent study revealed that the applicability of STOPP/START criteria to national databases decreased with each version [35]. Creating an effective CDSS implementing STOPP/START version 3 may be challenging. Thus, our study emphasizes valuable newly formed criteria with a prevalence of at least 5% within the studied population, which is essential for an effective future CDSS tool.

The study has several limitations. First, our sample size was limited, categorized as a convenience sample, and enabled partially dependent patients. Due to a lack of external validity, the results may not be generalizable to the rest of the population. The retrospective design of the study precludes the assessment of clinical outcomes resulting from PIP detected by STOPP/START criteria. Nevertheless, our study is the first which showed the prevalence of PIP as detected by STOPP/START version 3 criteria. Further studies should take into account a larger study group and prospective trials assessing clinical outcomes.

## 5. Conclusions

The study shows the high prevalence of PIP detected by STOPP/START version 3 criteria in ambulatory care geriatric patients in Poland. The new criteria were more efficient than the previous, second version, in both the STOPP and START sections. Several newly formed criteria identified PIP in a substantial proportion of the population. Criteria regarding NSAIDs, including aspirin in cardiovascular indications, were common PIMs identified by STOPP v3. Three newly formed START criteria (the lack of mineralocorticoid receptor antagonists and SGLT-2 inhibitors in heart failure and the lack of osmotic laxatives for chronic constipation) accounted for most of the additional omissions, compared with START v2. The moderate to strong concordance between PIP identified by the new and the previous version, together with the higher PIP detection rate, suggest that STOPP/START v3 is a relevant expansion of this tool.

## Figures and Tables

**Table 1:** Potentially inappropriate medications (PIMs) identified by STOPP v3 in comparison to STOPP v2. The criteria sections have been organized from the most numerous to the least numerous PIMs. Criteria with a prevalence of at least 5% were shown, along with a note indicating whether STOPP v2 includes them—“+” present in STOPP v2, “−“ not present in STOPP v2.

Criteria	STOPP v3	STOPP v2
**Coagulation system**	**38**	**14**
Aspirin for primary prevention of cardiovascular disease	11	−
Long-term aspirin at doses greater than 100 mg per day	10	−
**Central nervous system**	**36**	**27**
Benzodiazepines for ≥ 4 weeks	10	+
Acetylcholinesterase inhibitors with drugs that reduce heart rate	8	+
**Cardiovascular system**	**25**	**12**
Non-topical NSAIDs with a history of cardiovascular disease	6	−
Loop diuretic for hypertension with urinary incontinence	5	+
Antipsychotics with a history of cardiovascular disease	5	−
**Analgesics**	**18**	**14**
Use of regular opioids without concomitant laxative	14	+
**Musculoskeletal system**	**15**	**4**
Long-term opioids for osteoarthritis	11	−
**Falls**	**14**	**7**
Antidepressants in patients with recurrent falls	5	−
**Gastrointestinal system**	**13**	**13**
Drugs causing constipation in patients with constipation	13	+
**Urogenital system**	**8**	**2**
**Duplicate class**	**5**	**5**
**Renal system**	**3**	**2**
**Endocrine system**	**2**	**1**
**Respiratory system**	**1**	**1**
**Antimuscarinic**	**1**	**1**
**Total**	**179**	**103**

NSAIDs, nonsteroidal anti-inflammatory drugs.

**Table 2:** Comparison of the prevalence of PIMs detected by STOPP v3 and STOPP v2 and the prevalence of PPOs detected by START v3 and START v2 using the McNemar test. Agreement between STOPP v2/STOPP v3 and START v2/START v3 measured by Cohen’s Kappa.

		STOPP v2		χ^2^	*p*-Value	Kappa
		**PIMs**	**Non-PIMs**			
**STOPP v3**	**PIMs**	75.342%	24.658%	15.2	<0.0001	0.6
	**Non-PIMs**	3.704%	96.296%			
		**START v2**				
		**PPOs**	**Non-PPOs**			
**START v3**	**PPOs**	77.027%	22.973%	17.0	<0.0001	0.64
	**Non-PPOs**	0%	100%			

**Table 3:** Potential prescribing omissions (PPOs) identified by START v3 in comparison to START v2. Criteria sections have been organized from the most numerous to the least numerous PPOs. Criteria with a prevalence of at least 5% were highlighted, along with a note indicating whether START v2 includes them—“+” present in START v2, “−“ not present in START v2.

Criteria	START v3	START v2
**Cardiovascular system**	**63**	**38**
SGLT-2 inhibitors in heart failure	22	−
Mineralocorticoid receptor antagonist in heart failure	13	−
Statin therapy with a history of cardiovascular disease	10	+
ACE inhibitor with coronary artery disease	9	+
Beta-blocker with symptomatic coronary artery disease	6	+
**Gastrointestinal system**	**42**	**3**
Osmotic laxative for chronic constipation	30	−
Proton pump inhibitor with NSAID	6	−
**Urogenital system**	**17**	**17**
5-alpha reductase inhibitor for lower urinary tract symptoms related to benign prostatic hyperplasia	9	+
Alpha-1 blocker for lower urinary tract symptoms related to benign prostatic hyperplasia	8	+
**Musculoskeletal system**	**15**	**15**
Bisphosphonates, vitamin D, and calcium with corticosteroid	7	+
**Analgesics**	**14**	**14**
Laxatives in patients receiving opioids	14	+
**Central nervous system**	**7**	**7**
Non-TCA antidepressant drug for major depression	6	+
**Coagulation system**	**6**	−
**Respiratory system**	**3**	**3**
**Endocrine system**	**0**	**0**
**Renal system**	**0**	−
**Total**	**167**	**95**

ACE, angiotensin-converting enzyme; SGLT-2, sodium–glucose co-transporter-2; Non-TCA, non-tricyclic; NSAID, nonsteroidal anti-inflammatory drug.

**Table 4:** Association between participants with PIMs or PPOs according to STOPP v3, STOPP v2, START v3, and START v2 criteria and their characteristics.

		Gender, Male	CCI	Cognitive Impairment
**STOPP v3**	**OR (95% CI)**	1.04 (0.41–2.65)	1.19 (0.88–1.61)	0.86 (0.31–2.42)
	***p*** **-value**	0.94	0.25	0.77
**START v3**	**OR (95% CI)**	0.21 (0.04–1.28)	**1.79 (1.19–2.68)**	1.28 (0.39–4.24)
	***p*** **-value**	0.93	**<0.01**	0.69
**STOPP v2**	**OR (95% CI)**	1.51 (0.65–3.48)	0.91 (0.71–1.17)	1.22 (0.49–3.05)
	***p*** **-value**	0.34	0.46	0.67
**START v2**	**OR (95% CI)**	0.83 (0.35–1.96)	1.13 (0.87–1.46)	1.91 (0.73–4.95)
	***p*** **-value**	0.67	0.36	0.19

CCI, Charlson Comorbidity Index; OR, odds ratio; CI, confidence intervals 95%.

## Data Availability

The study datasets are available from the corresponding author at reasonable request.

## Supplementary Materials

The following supporting information can be downloaded at: https://www.mdpi.com/article/10.3390/jcm13206043/s1, **Table S1**: The list of unused STOPP/START criteria due to a lack of specific data.

## Abbreviations

PIP, potentially inappropriate prescribing; MAI, Medication Appropriateness Index; FORTA, Fit fOR The Aged; STOPP, Screening Tool of Older Person’s Prescriptions; START, Screening Tool to Alert doctors to Right Treatment; PILA, patient-in-focus listing approach; DOLA, drug-oriented listing approach; DOLA+, drug-oriented listing approach but indication required; PIMs, potentially inappropriate medications; PPOs, potential prescribing omissions; IQR, interquartile range; SD, standard deviation; CNS, central nervous system; NSAIDs, nonsteroidal anti-inflammatory drugs; v3, version 3; v2, version 2; SGLT-2, sodium–glucose co-transporter-2; ACE, angiotensin-converting enzyme; SARS-CoV2, severe acute respiratory syndrome coronavirus 2.
