# Supplementary material for: Potentially Inappropriate Prescribing Identified Using STOPP/START Version 3 in Geriatric Patients and Comparison with Version 2: A Cross-Sectional Study
Source: J Clin Med. 2024 Oct 10;13(20):6043. doi: 10.3390/jcm13206043 (PMC11508511; doi:10.3390/jcm13206043)
Supplement: Supplementary file 1 [file jcm-13-06043-s001.zip › jcm-3198147-supplementary.pdf]

Unused STOPP/START criteria from version 3			
PART	SECTION	CRITERIA	
STOPP	Indication of medication	A1	Any drug prescribed without a clinical indication.
		A2	Any drug prescribed beyond the recommended duration, where the treatment duration is well defined.
	Cardiovascular system	B13	Aldosterone antagonists (e.g., spironolactone, eplerenone) with concurrent potassium-conserving drugs (e.g., ACEI's, ARB's, amiloride, triamterene) without monitoring of serum potassium (risk of dangerous hyperkalemia, i.e., > 6.0 mmol/l—serum K should be monitored regularly, i.e., at least every 6 months).
		B15	Drugs that predictably prolong the QTc interval (QTc = QT/RR) in patients with known with demonstrable QTc prolongation (to >450 msec in males and >470 msec in females), including quinolones, macrolides, ondansetron, citalopram (doses of > 20 mg/day), escitalopram (doses of > 10 mg/day), tricyclic antidepressants, lithium, haloperidol, digoxin, class 1A antiarrhythmics, class III antiarrhythmics, tizanidine, phenothiazines, astemizole, and mirabegron (risk of life-threatening ventricular arrhythmias).
		B16	Statins for primary cardiovascular prevention in persons aged ≥ 85 experiencing established frailty, with a life expectancy of likely less than 3 years (lack of evidence of efficacy).
	Central nervous system	D5	Antipsychotics prescribed for behavioral and psychological symptoms of dementia (BPSD), at an unchanged dose for > 3 months without medication review (an increased risk of extrapyramidal side effects and chronic worsening of cognition, as well as an increased risk of major cardiovascular morbidity and mortality).
		D15	Antipsychotics in patients with behavioral and psychological symptoms of dementia (BPSD) for longer than 12 weeks, unless BPSD symptoms are severe and other nonpharmacological treatments have failed (increased risk of stroke, myocardial infarction).
	Musculoskeletal system	H3	Long-term use of NSAID (>3 months) for the relief of osteoarthritis pain as a symptom, where paracetamol has not been tried (simple analgesics are preferable and are usually as effective for pain relief).
	Analgesic drugs	L1	Use of strong oral or transdermal opioids (morphine, oxycodone, fentanyl, buprenorphine, diamorphine, methadone, tramadol, pethidine, pentazocine) as first-line therapy for mild pain (WHO analgesic ladder not observed; paracetamol or NSAID not prescribed as first-line therapy).
		L3	Long-acting opioids without short-acting opioids for break-through moderate or severe pain (risk of persistence of severe pain).
START	Indicated drugs	A1	Where a drug is clearly indicated and considered appropriate in the particular clinical context and there is no clear contraindication, that drug should be initiated as per formulary guidelines for dose and duration.
	Cardiovascular system	B9	Sacubitril/valsartan in heart failure with reduced ejection fraction, causing persistent heart failure symptoms, despite optimal dose of ACE inhibitor or angiotensin receptor blocker (sacubitril/valsartan to replace ACE inhibitor or angiotensin receptor blocker).
		B10	Beta-blocker for chronic atrial fibrillation with uncontrolled heart rate.
	Renal system	E2	Phosphate binder in severe chronic kidney disease (i.e., eGFR < 30 ml/min/m2) if the serum phosphate concentration is persistently >1.76 mmol/l (5.5 mg/dl), despite adherence to a renal diet.
		E4	Angiotensin receptor blocker (ARB) or angiotensin converting enzyme inhibitor (ACE-I) in chronic kidney disease with proteinuria, i.e., urine albumin excretion of >300 mg/24 hours.
	Respiratory system	G2	Regular, i.e., daily inhaled corticosteroid (e.g., beclomethasone, budesonide, ciclesonide, fluticasone, mometasone) for moderate–severe asthma or COPD of GOLD 3 or 4 severity, where FEV1 is <50% of the predicted value and repeated exacerbations are present, requiring treatment with oral corticosteroids.
	Musculoskeletal system	H5	Vitamin D supplement in older people with confirmed 25-hydroxycolecalciferol deficiency (< 20 micrograms/L, < 50 nmol/L) who are housebound or experiencing falls or with osteopenia (with a bone mineral density T-score of less than -1.0 but above -2.5 in one or multiple sites).
		H6	Anti-resorptive treatment after the discontinuation of at least two doses of denosumab (rebound increased bone turnover markers, BMD loss, and increased risk of vertebral fracture following denosumab discontinuation).
		H7	Anti-resorptive treatment after the discontinuation of teriparatide/abaloparatide treatment for osteoporosis.
	Analgesics	K1	High-potency opioids in moderate–severe pain, where paracetamol, NSAIDs, or low-potency opioids are not appropriate for the pain severity or have been ineffective.
	Vaccines	L3	Varicella-zoster vaccine, according to national guidelines.

ACEI, ACE-I, angiotensin-converting enzyme inhibitors; ARB, angiotensin receptor blocker; QTc, QT interval corrected for heart rate; QT, QT interval; RR, interval from the onset of one QRS complex to the onset of the next QRS complex; BPSD, behavioral and psychological symptoms of dementia; WHO, World Health Organization; NSAID, nonsteroidal anti-inflammatory drug; ACE, angiotensin-converting enzyme; eGFR, estimated glomerular filtration rate; COPD, chronic obstructive pulmonary disease; GOLD, The Global Initiative for Chronic Obstructive Lung Disease; FEV1, forced expiratory volume in one second; BMD, bone mineral density.

Not used STOPP/START criteria from version 2			
PART	SECTION	CRITERIA	
STOPP	Indication of medication	A1	Any drug prescribed without an evidence-based clinical indication.
		A2	Any drug prescribed beyond the recommended duration, where the treatment duration is well defined.
	Cardiovascular system	B12	Aldosterone antagonists (e.g., spironolactone, eplerenone) with concurrent potassium-conserving drugs (e.g., ACEIs, ARB's, amiloride, triamterene) without monitoring of serum potassium (risk of dangerous hyperkalemia, i.e., > 6.0 mmol/l – serum K should be monitored regularly, i.e., at least every 6 months).
	Central nervous system and psychotropic drugs	D9	Neuroleptic antipsychotic in patients with behavioral and psychological symptoms of dementia (BPSD) unless symptoms are severe and other non-pharmacological treatments have failed (increased risk of stroke).
	Musculoskeletal system	H3	Long-term use of NSAID (>3 months) for relief of osteoarthritis pain as a symptom, where paracetamol has not been tried (simple analgesics are preferable and are usually as effective for pain relief).
	Analgesic drugs	L1	Use of strong oral or transdermal opioids (morphine, oxycodone, fentanyl, buprenorphine, diamorphine, methadone, tramadol, pethidine, pentazocine) as first-line therapy for mild pain (WHO analgesic ladder not observed).
		L3	Long-acting opioids without short-acting opioids for break-through pain (risk of persistence of severe pain).
START	Respiratory system	B2	Regular inhaled corticosteroid for moderate–severe asthma or COPD, where FEV1 is <50% of the predicted value and repeated exacerbations are present, requiring treatment with oral corticosteroids.
	Central nervous system and eyes	C4	Topical prostaglandin, prostamide, or beta-blocker for primary open-angle glaucoma.
	Musculoskeletal system	E5	Vitamin D supplement in older people who are housebound or experiencing falls or experiencing osteopenia (with a bone mineral density T-score of > -1.0 but < -2.5 in multiple sites).
	Endocrine system	F1	ACE inhibitor or angiotensin receptor blocker (if intolerant of ACE inhibitor) in diabetes with evidence of renal disease, i.e., dipstick proteinuria or microalbuminuria (>30mg/24 hours) with or without serum biochemical renal impairment.
	Analgesics	H1	High-potency opioids in moderate–severe pain, where paracetamol, NSAIDs, or low-potency opioids are not appropriate for the pain severity or have been ineffective.

ACEI, angiotensin-converting enzyme inhibitors; ARB, angiotensin receptor blocker; BPSD, behavioral and psychological symptoms of dementia; NSAID, nonsteroidal anti-inflammatory drug; WHO, World Health Organization; COPD, chronic obstructive pulmonary disease; FEV1, forced expiratory volume in one second; ACE, angiotensin-converting enzyme.
